# Independent working memory resources for egocentric and allocentric spatial information

**DOI:** 10.1371/journal.pcbi.1006563

**Published:** 2019-02-21

**Authors:** David Aagten-Murphy, Paul M. Bays

**Affiliations:** Department of Psychology, University of Cambridge, Cambridge, United Kingdom; UC Davis, UNITED STATES

## Abstract

Visuospatial working memory enables us to maintain access to visual information for processing even when a stimulus is no longer present, due to occlusion, our own movements, or transience of the stimulus. Here we show that, when localizing remembered stimuli, the precision of spatial recall does not rely solely on memory for individual stimuli, but additionally depends on the relative distances between stimuli and visual landmarks in the surroundings. Across three separate experiments, we consistently observed a spatially selective improvement in the precision of recall for items located near a persistent landmark. While the results did not require that the landmark be visible throughout the memory delay period, it was essential that it was visible both during encoding and response. We present a simple model that can accurately capture human performance by considering relative (allocentric) spatial information as an independent localization estimate which degrades with distance and is optimally integrated with egocentric spatial information. Critically, allocentric information was encoded without cost to egocentric estimation, demonstrating independent storage of the two sources of information. Finally, when egocentric and allocentric estimates were put in conflict, the model successfully predicted the resulting localization errors. We suggest that the relative distance between stimuli represents an additional, independent spatial cue for memory recall. This cue information is likely to be critical for spatial localization in natural settings which contain an abundance of visual landmarks.

## Author summary

Human capacity to maintain spatial information over brief interruptions is strongly limited. However, while studies of visual working memory typically examine recall in sparse displays, consisting only of the stimuli to remember, natural scenes are commonly filled with other objects that—although not required to be remembered—may nevertheless influence subsequent localization. We demonstrate that memory for spatial location depends on independent stores for egocentric (relative to the observer) and allocentric (relative to other stimuli) information about object position. Both types of spatial representation become increasingly imprecise as the number of objects in memory increases. However, even when visual landmarks are present—and allocentric information encoded—there is no change in egocentric precision. This suggests that the encoding of additional allocentric spatial information does not compete for working memory resources with egocentric spatial information. Additionally, the fidelity of allocentric position information diminished rapidly with distance, resulting in a spatially specific advantage for recall of objects in the vicinity of stable landmarks. The effect of a landmark on recall matches that of an ideal observer who optimally combines egocentric and allocentric cues. This work provides a new experimental and theoretical framework for the investigation of spatial memory mechanisms.

## Introduction

Imagine trying to locate your friends while watching a crowded street parade. If you catch only a glimpse of them in the crowd before they are obscured by others, remembering how far they were from a nearby building (a stable landmark in the external world) may provide a useful cue to help you localize them later. Indeed, this relative (or allocentric) information may prove more valuable than memory of their location within your visual field (egocentric information). However, the nature of storage of allocentric information, and its interaction with other forms of visual memory, have not been clearly established.

Interruptions in sensory input represent a frequent challenge to the visual system, whether due to our own actions, such as an eye-movement or blink, or changes in the external world, such as object occlusions or the disappearance of a transient stimulus. Visuospatial working memory (VSWM) helps bridge these discontinuities, by allowing us to retain sensory information about visual objects even when they are no longer visible. However, the capacity of VSWM to store information is limited. Even when explicitly instructed to remember specific stimuli—in anticipation of an interruption—individuals make substantial errors in both their ability to detect the occurrence of a change [1–3] and to reproduce remembered features [4,5]. Error increases monotonically as the number of items increases, and this holds true for recall of object locations as well as features [6]. This is consistent with models in which objects compete for allocation of a limited representational resource [4,7–9].

Representations of visual information in early visual cortex are inherently egocentric, emerging directly from the projection of the external world onto the retina. Consequently, the spatial information associated with visual processing is at least initially gaze-centered, encoding locations relative to the observer, and decreasing in resolution as the distance from the fovea increases [10]. This retinotopic spatial encoding appears to be preserved throughout much of the brain, particularly in dorsal brain regions that support the execution of actions towards remembered locations [11,12] (but see [13,14] with respect to ventral areas). Indeed, it is actively debated whether spatial information is ever encoded in non-retinotopic reference frames [15–18]. The point of contention is whether separable representations of stimuli are encoded—within distinct neural populations and potentially within different neural pathways—or if the apparent use of other representations merely reflects timely manipulations of egocentric information [16]. Important evidence has come from studies of motor action, which have shown that movement errors are reduced in the presence of visual landmarks, and suggested motor programming reflects the combination of egocentric (retinotopically encoded relative to current gaze) and allocentric (relative to external landmarks) spatial cues [19–21].

In this paper we investigate how egocentric and allocentric VSWM representations interact. Specifically, in view of the limited capacity of VSWM, we examine the impact of encoding additional allocentric spatial information in the form of distance from a visual landmark. We show that the behavioral data is consistent with an optimal integration of an egocentric signal, independent of the landmark, with an allocentric signal that degrades with distance from the landmark. We further show that allocentric information does not compete with egocentric information for storage, indicating that the two sources of information rely on independent memory resources.

## Results

### A stable landmark enhances spatial working memory precision

**Fig 1:**
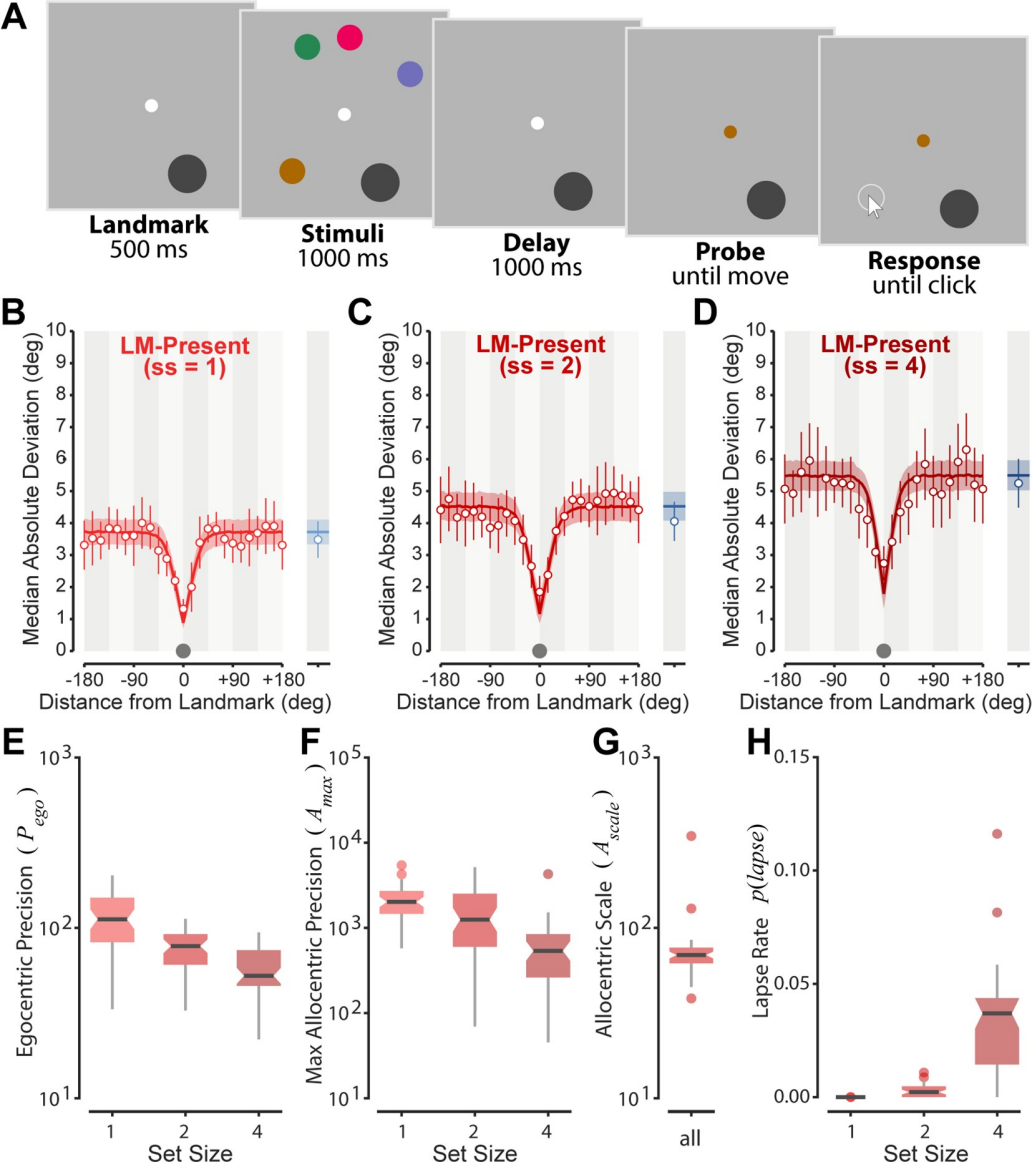
Experiment one. **(A)** LM-PRESENT design. Participants memorized the locations of colored disks in the presence of a landmark (a larger dark gray disk; note object sizes are exaggerated for visibility). **(B-D)** Data points indicate mean variability in location recall for set sizes 1, 2 and 4 respectively, with predictions of the optimal integration model overlaid (colored lines). Note the model captures both the reduction in variability near the landmark, and the plateau in variability at far landmark-target separations. LM-PRESENT data is shown in red, LM-ABSENT in blue. Errorbars and patches indicate 95% CI. Gray dots indicate size of the landmark on the x-axis scale. **(E-H)** Box plots depicting parameter estimates for the best-fitting model (notch represents 95% confidence interval on the median). Note the decrease in egocentric precision (E), decrease in allocentric precision (F) and increase in lapse rate (H) associated with increasing set size, while the best-fitting model exhibited no changes in the allocentric scale (rate of decay with distance), which is therefore estimated by a single parameter (G).

In Experiment 1 we investigated the influence of a visual landmark on spatial working memory for different numbers of remembered objects (set size: 1, 2 or 4). Participants used a computer mouse to report the remembered location of one item from a memory array, identified by color (Fig 1A). Examining spatial recall precision in the absence (LM-ABSENT) and presence (LM-PRESENT) of a stable visual landmark, we observed a substantial reduction in the variability of memory reproduction for stimuli located near the landmark, at all set sizes (Fig 1B–1D). These changes occurred in the absence of systematic shifts in bias (S3 Fig) and indicate that the presence of the landmark gave participants access to additional information to facilitate recall.

**Fig 2:**
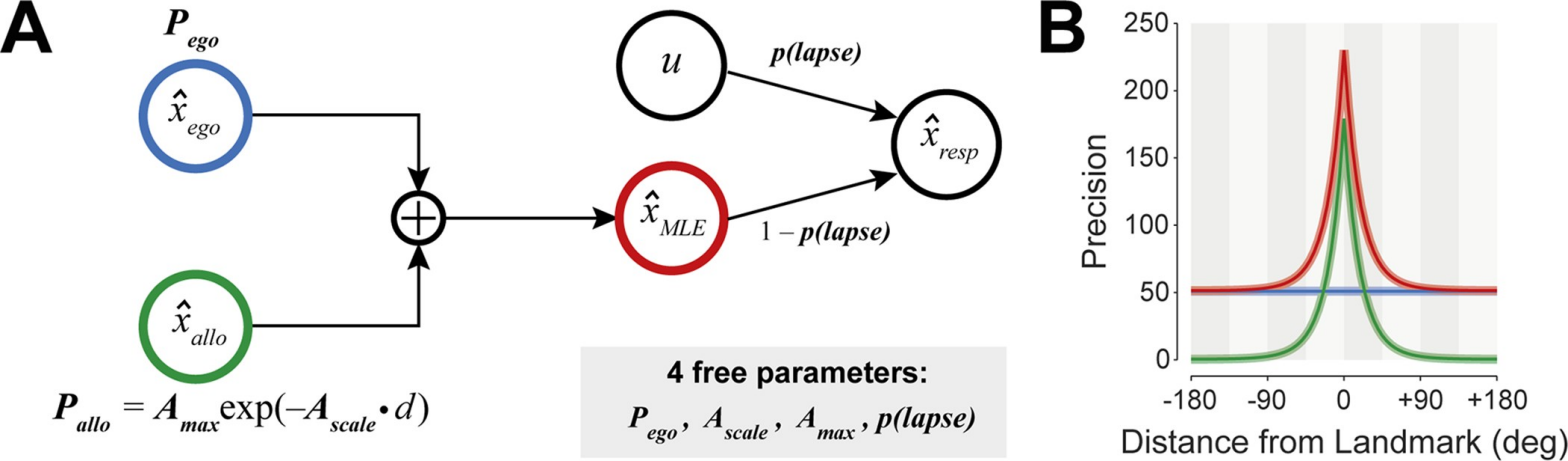
Ideal observer model. **(A)** The visual working memory decoding model, in which egocentric and allocentric estimates are integrated depending on their respective reliabilities. While precision of the egocentric component is set by *P*_*ego*_, the allocentric precision is determined by two parameters: the peak precision obtained when landmark and target are aligned (*A*_*max*_), and a scale parameter describing how quickly allocentric precision declines with increasing landmark-target distance (*A*_*scale*_). The model further incorporates a fixed probability of lapsing (*p(lapse);* responding at random relative to the target), giving four free parameters in total. **(B)** Precision of egocentric (blue) and allocentric (green) estimates shown as a function of distance from the landmark. While egocentric precision is constant, the precision of allocentric information decreases exponentially as the distance increases. The precision of the integrated estimate (red) is equal to the sum of precisions of the individual components.

We implemented a simple cue-combination model to investigate whether this spatially selective improvement in precision could be captured by optimal integration of independent *egocentric* and *allocentric* spatial encodings (Fig 2; see Methods). In the model, the precision of the allocentric signal diminishes with distance to the landmark from a peak A_*max*_ at rate A_*scale*_, while precision of the egocentric signal is independent of distance. The model also includes a lapse rate to capture random responding and “swap” errors [4,9]. The fit of the optimal integration model is shown as solid lines in Fig 1B–1D. This model provided a substantially better fit to data than a reduced model with allocentric encoding omitted (ΔAICc = 662).

Consistent with previous studies [4,5,22], the precision of the egocentric signal declined with increasing set size (Fig 1E; comparison to model with fixed precision: ΔAICc = 234; linear regression slope = –18.4 ± 2.7 (M ± SE), t(11) = 6.89, p < 0.001). Similarly, model comparison indicated a decrease in peak precision of the allocentric signal with set size (Fig 1F; ΔAICc = 45.94; linear regression slope = -461 ± 78; t(11) = 5.88; p < 0.001). There were no changes across set size in the rate with which precision of the allocentric signal scaled with distance (Fig 1G; ΔAICc = 18.45). The lapse rate increased with set size but accounted for only a very small fraction of trials (Fig 1H; ΔAICc = 139; slope = 0.014 ± 0.003; t(11) = 4.48; p < 0.001).

### Independence of egocentric and allocentric stores

The presence of a visual landmark substantially improved the localization of memory stimuli in the landmark’s vicinity, implying that participants remembered the allocentric distance between the landmark and each memory stimulus, in addition to the egocentric location of each stimulus. In previous studies, increasing the amount of information stored in working memory has consistently been shown to decrease the precision of recall, consistent with distribution of a limited memory resource between items to be remembered [7,9]. If egocentric and allocentric encodings of location similarly share memory resources, the additional inclusion of relative information should convey a cost in the form of decreased precision of egocentric information. However, we found no evidence for such a cost, as can be seen qualitatively in Fig 1A–1C by comparing LM-PRESENT performance at 180° separation to LM-ABSENT performance. Were there a cost in the fidelity of egocentric information associated with encoding allocentric information, then localization of targets far from the landmark (where allocentric information should make a negligible contribution to response precision) would be noticeably more variable than in the absence of a landmark. A model in which egocentric precision decreased in the presence of a landmark (see Methods) provided a substantially worse description of the data (ΔAICc = 53.65), confirming that memory resources for egocentric and allocentric information are independent. The distribution of individual parameter values obtained for the rejected model (in this and subsequent experiments) was also inconsistent with a cost to egocentric precision (see S2 Text and S2 Fig).

### Landmark persistence

**Fig 3:**
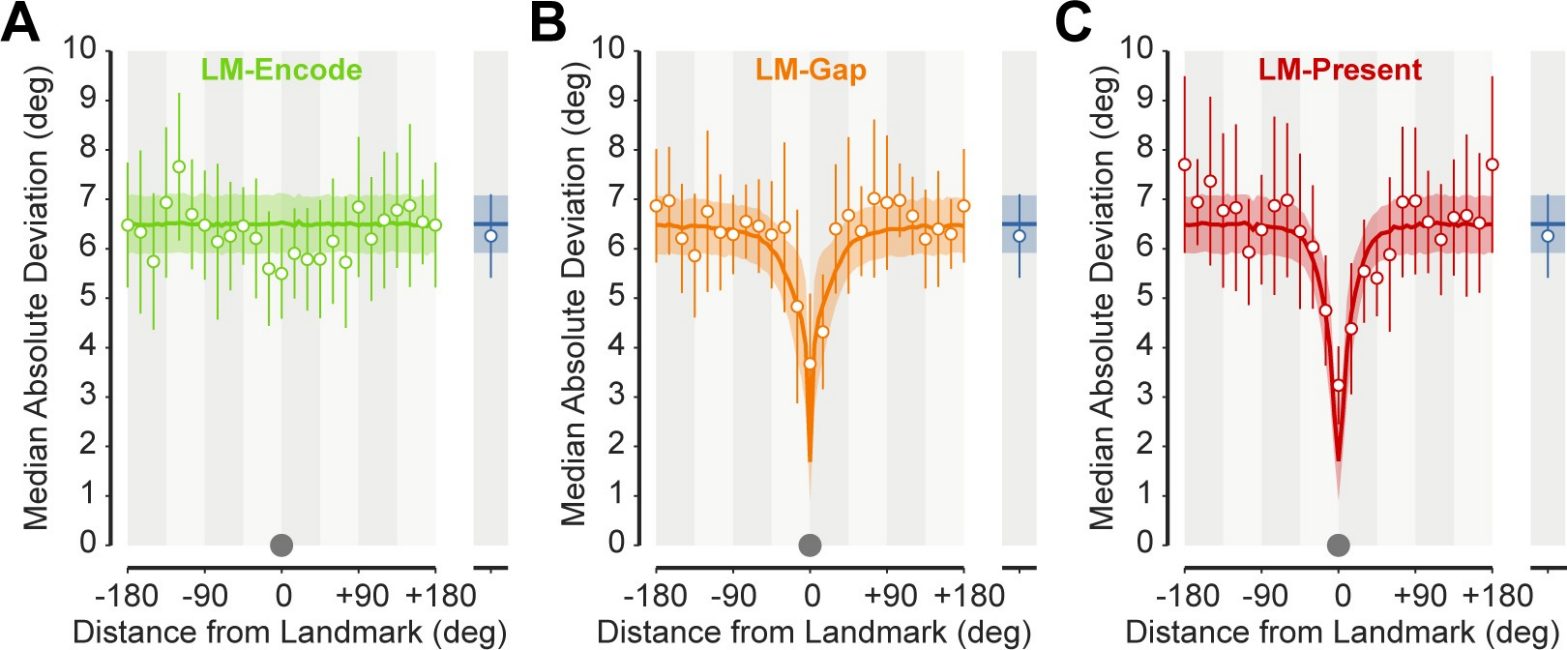
Experiment two. **(A-C)** Mean variability in memory recall across participants for LM-ENCODE (A), LM-GAP (B) and LM-PRESENT (C) conditions (with LM-ABSENT shown on the right in blue). There is a substantial reduction in variability in the vicinity of the landmark irrespective of whether the landmark was persistently (LM-PRESENT) or intermittently shown (LM-GAP), but no apparent influence of the visual landmark when it was only visible during encoding (LM-ENCODE). Predictions of the best-fitting model are overlaid.

A plausible alternative account of the landmark effect is that the presence of the salient landmark in the initial array biased encoding towards memoranda in its vicinity. In Experiment 2 we tested a condition (LM-ENCODE) in which the landmark was visible only during the presentation of the memory array (set size 4). This condition was interleaved with other conditions such that participants did not know during encoding whether the landmark would disappear. We observed no landmark-related improvement of precision in this condition (Fig 3A) and a reduced model with no allocentric signal provided a better fit to data than the optimal integration model (ΔAICc = 16.56). This confirms that the landmark benefit is a result of the use of allocentric spatial information and not due to encoding bias. If items presented in the vicinity of a landmark were preferentially encoded, or encoded with enhanced precision, we would have seen a benefit for those items even when the landmark was absent during the response phase.

Does the use of the landmark depend on its continuous presence during the memory delay? We tested a condition (LM-GAP) in which the landmark disappeared at the offset of the sample array and only reappeared at the time of the probe. We found a robust landmark effect in this condition (ΔAICc = 135 compared to reduced model; Fig 3B). Comparing the LM-GAP condition to one in which the landmark was continuously present (LM-PRESENT, as in Exp 1) revealed no difference in peak precision of the allocentric signal (ΔAICc = 9.34 favoring a model with shared *A*_*max*_ parameter between conditions) but some evidence for a difference in the rate of change of precision with distance (ΔAICc = 13.19 favoring a model in which *A*_*scale*_ differed between conditions; median *A*_*scale*_ 20.9% lower in LM-GAP condition). Exp 2 also replicated the finding from Exp 1 that the presence of a landmark incurred no cost to the precision of egocentric memory (model with cost performed worse, ΔAICc = 23.31; parameter estimates for Exp 2 are shown in S4 Fig).

A final possibility is that the benefits observed in the LM-GAP and LM-PRESENT conditions arose from enhanced retrieval of items whose previous locations were close to the landmark’s location at the time of the probe, perhaps due to internal attention being drawn to that location in memory. We therefore carried out an additional control experiment (see S1 Text and S1 Fig) which included an LM-RETRIEVE condition, in which the landmark was visible only at the time of response, and not during the presentation of the memory stimuli. In this condition, allocentric information about the items’ locations relative to the landmark could not be encoded from the memory array, but any effect of the landmark on internal attention at the time of retrieval should still be present. We found no evidence for a landmark-related improvement of precision in this condition, and a reduced model with no allocentric signal for the LM-RETRIEVE condition provided a better fit to data than the optimal integration model (ΔAICc = 39.86).

Considering in combination the results of LM-ENCODE (no benefit if the landmark is present only during encoding), LM-RETRIEVE (no benefit if the landmark is present only during retrieval) and LM-GAP (clear benefit if the landmark is present during both encoding and retrieval), our results strongly indicate that landmark-related benefits are due to encoding and subsequent retrieval of allocentric (relative position) information present in the memory array.

### Cue conflict

**Fig 4:**
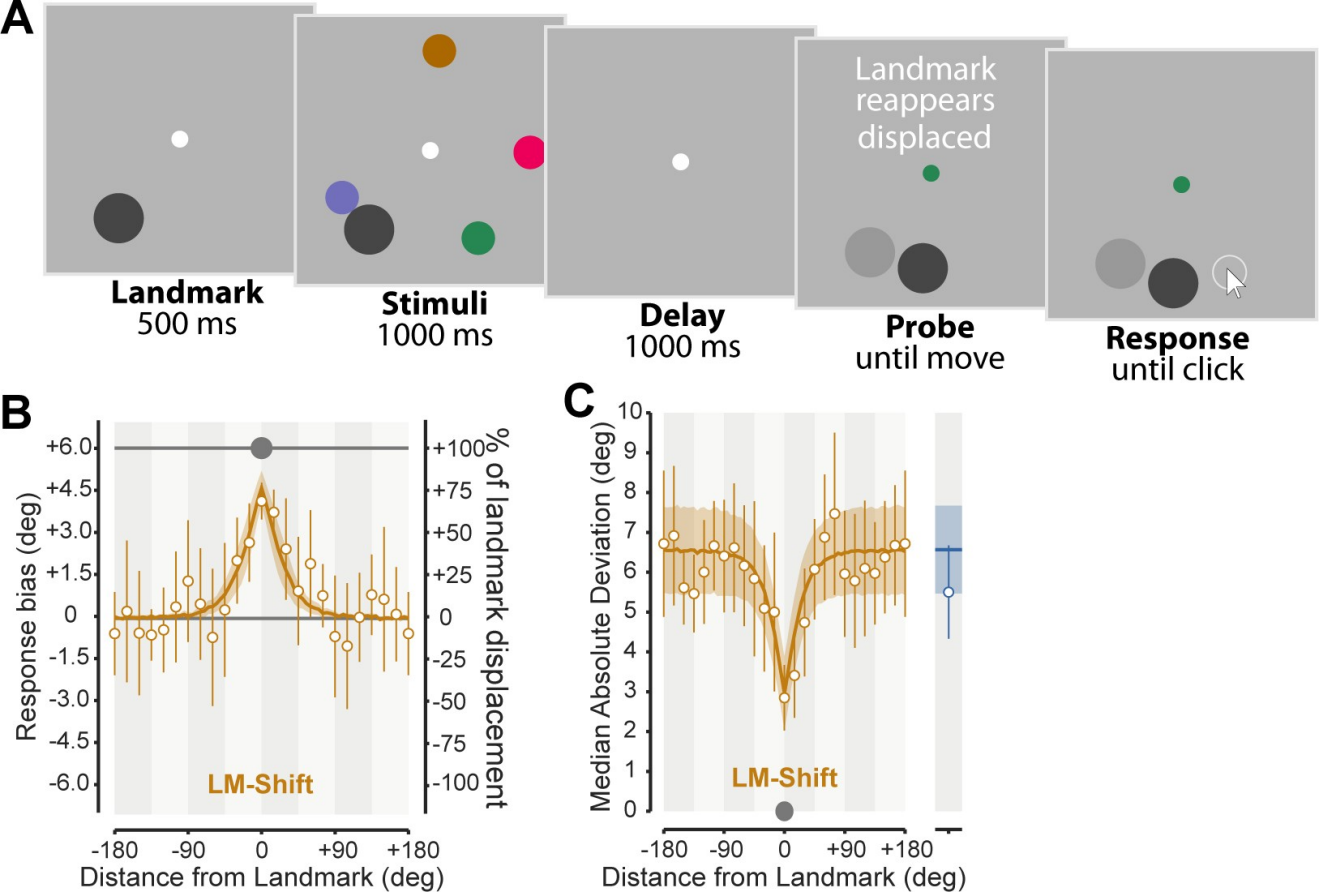
Experiment three. **(A)** Example LM-SHIFT trial. When the landmark returned, it was shifted by either 6° clockwise or counter-clockwise (exaggerated above for clarity; light gray disk illustrates previous landmark location and was not visible in the experiment). If participants used the post-shift location to anchor their allocentric estimates, we would expect their responses to be biased in the direction of the displacement, with the magnitude related to the reliability of the allocentric cue. **(B)** The response bias measured in the direction of the shift (magnitude 6° indicated by gray line), as a function of distance from the landmark. The data reveals a consistent bias in the direction of the displacement, which may be either towards or away from the visible landmark location. Bias magnitude depended on distance from the landmark with a peak of ~80% of the shift. **(C)** Spatially specific decreases in response variability near the landmark in LM-SHIFT. Note that for clarity the bias was subtracted prior to calculation of the median absolute deviation. The model predictions (overlaid) simultaneously capture landmark effects on both bias (B) and variability (C), without any additional free parameters.

To provide a strong test of the optimal integration model, in Experiment 3 we implemented a variant of the LM-GAP condition in which the landmark reappeared at a location displaced through a small distance (6° on the circle) from its original position (LM-SHIFT; Fig 4A). According to the model, this manipulation should introduce a conflict between egocentric and allocentric spatial information, with the allocentric estimate shifting with the visual landmark. As a result, we predicted that participants would show systematic biases in their localization responses in the direction of the shift, with the strength of the bias determined by the relative reliability of each cue.

We found a clear landmark effect in the LM-SHIFT condition (ΔAICc = 179 compared to reduced model), and the recalled locations of items presented close to the landmark were strongly shifted in the direction of landmark displacement (Fig 4B). The optimal integration model accurately predicted changes in both bias and variability with landmark distance (Fig 4B&4C). The additional fitting of bias required no extra parameters, relying on the same reliability estimates used to calculate variability. Parameter estimates for Exp 3 are shown in S5 Fig.

Examining the effect of shifting the landmark on precision of the allocentric signal (by contrasting LM-GAP and LM-SHIFT conditions), revealed a reduction in both the peak precision of allocentric information (ΔAICc = 19.03 favoring a model in which *A*_*max*_ differed between conditions; median *A*_*max*_ 74.0% lower in LM-SHIFT condition) and the rate at which it decayed with distance (ΔAICc = 21.6 favoring a model in which *A*_*scale*_ differed between conditions; median *A*_*scale*_ 35.1% lower in LM-SHIFT condition).

Finally, as in previous experiments, we examined whether there was evidence for a precision cost on egocentric encoding. We found a ΔAICc of 28.11 favoring the model without cost, further confirming that allocentric and egocentric information are independently stored.

## Discussion

Natural scenes rarely contain only a single item, and are instead frequently populated by multiple stable objects, any of which could act as a visual landmark for locations we need to remember. However, how the brain stores and uses this information is only partially understood. Here, using simple experimental displays, we have demonstrated a spatially specific enhancement of localization precision in the vicinity of a landmark, consistent with observers using not only memory of the egocentric spatial locations of stimuli, but also memory of their locations relative to other objects in the environment (allocentric information). We further investigated the consequences of encoding this additional information into VSWM, in light of established limitations on working memory resources [4,7,9,22].

It is now well established that increasing the number of items to be remembered increases variability in recall of their features and locations [4–6,22,23]. If egocentric and allocentric information compete for access to the same limited memory resource, then the introduction of a landmark (with the consequent encoding of additional allocentric information) should reduce egocentric memory fidelity. While we observed a spatially specific increase in recall precision for items near the landmark, memory items located far from the landmark were recalled just as precisely as when the landmark was absent. Thus, our results demonstrate that the presence of the landmark had no influence on the fidelity of egocentric memory representation. Instead, the presence of a landmark appeared to grant access to an additional allocentric source of spatial information. To confirm this finding, we incorporated a cost parameter into a cue-combination model, which allowed the reliability of egocentric information to be degraded in the presence of a landmark. In three separate experiments, we consistently found a model with no cost provided the best description of the data, a result further supported by a meta-analysis of cost estimates pooled across experiments (S2 Text and S2 Fig). Thus, rather than directly competing, our results suggest that egocentric and allocentric locations are encoded independently and draw upon separate memory resources.

We also examined competition *within* each representation as the number of items encoded increased. For egocentric spatial information, this competition led to a gradual decrease in the reliability of spatial estimates (*P*_*ego*_) as set size increased, consistent with previous results [6,7]. Similarly, we found that increasing set size led to a decrease in the maximum reliability of allocentric spatial information (*A*_*max*_), indicating that the recollection of multiple relative locations also reflects a distribution of limited memory resources. In contrast, set size had no influence on the rate at which allocentric precision diminished with distance (*A*_*scale*_). So, while the number of items in memory determined the overall reliability of allocentric information, the relationship between landmark distance and reliability appears to be fixed.

We observed a substantial, spatially specific improvement in recall precision even when the landmark was hidden during the memory delay (LM-GAP). While this manipulation did not change the maximum precision of allocentric information (*A*_*max*_), there was a decrease in the spatial scale over which the precision enhancement was observed (*A*_*scale*_). The interruption in landmark persistence may have reduced the perceived stability of the visual landmark, introducing uncertainty as to whether the returning landmark had reappeared at the same location or if it should be considered the same object [24–27]. This is consistent with a study of reach programming in which landmark locations were jittered [19], which demonstrated that participants are sensitive to perceived landmark stability and adjust reliance on allocentric information as a result.

Importantly, when the landmark was only present during encoding (LM-ENCODE)—and not during recall—there was no advantage in localization compared to conditions without a landmark (LM-ABSENT). This means the landmark benefit cannot be explained simply by enhanced encoding of items in its vicinity, as this would predict improved localization irrespective of the landmark’s presence at recall. Furthermore, because the interleaved LM-GAP and LM-ENCODE conditions were indistinguishable until the time of recall, we can be certain that the same amount of allocentric information was encoded in both conditions. Therefore, the absence of a benefit in LM-ENCODE must arise from an inability to use this information. A landmark only appears to improve localization performance when at the time of testing the recalled allocentric distance can be anchored to the visible location of the landmark itself.

We also saw no benefit when the landmark was present only at the time of retrieval (LM-RETRIEVE), a condition in which allocentric information relating memory items to the landmark could not have been stored. This also demonstrates that the landmark benefit is not due to an enhancement of retrieval for items whose location in memory falls close to the probe, as such an enhancement would be observed regardless of whether the landmark was visible during encoding.

The lack of difference in recall precision between LM-ENCODE and LM-ABSENT conditions enables two additional observations to be made. First, participants apparently did not encode the egocentric location of the visual landmark itself, as its presence had no influence on precision (i.e. there was no set-size effect diminishing precision in the LM-ENCODE condition). This is consistent with both the task instructions and our conclusion that, to be useful for localization, the landmark had to be present at test. Second, given that we know allocentric information was encoded (but not used) in the LM-ENCODE condition, any competition between allocentric and egocentric information would be readily apparent as a decrease in precision compared to LM-ABSENT. The absence of such a difference is itself strong additional evidence for the independence of egocentric and allocentric spatial representations.

Across a variety of different conditions, an optimal integration model accurately described how allocentric and egocentric information were combined to generate estimates of location. Based on exponential decay of allocentric precision with distance from the landmark, this model captured not only how recall variability changed as a function of distance, but also the distance-dependent recall biases that emerged when egocentric and allocentric cues were put in conflict (Exp 3). Specifically, when we covertly changed the location at which the landmark reappeared (LM-SHIFT), we observed systematic shifts in recall position based on the distance of the recalled item from the landmark, with memoranda near to the landmark biased substantially in the direction of the displacement. Critically, these biases were consistent with a displacement in localization (i.e. relying more strongly on the allocentric information), not with an attractive bias to the landmark’s location. This result adds considerable support for our model, demonstrating that the integration of egocentric and allocentric information was close to optimal, and reinforces the conclusion that allocentric and egocentric estimates are encoded separately and as such associated with independent noise. Such integration models have proved invaluable in the study of multisensory integration (e.g. [28,29]), and several studies have used similar methods to describe the integration of allocentric and egocentric information in reaching and eye-movements to a single target [19–21,30,31]. However, to our knowledge, no previous study has quantitatively examined the consequences of encoding both egocentric and allocentric information on memory fidelity, determined how the precision of allocentric spatial information varies with set size, nor quantified the relationship between distance and the reliability of allocentric information.

The results of the LM-SHIFT condition also provide evidence against any alternative account of our findings based on local changes in the encoding or retrieval of items in the vicinity of the landmark. The observed biases in recall could not be the result of a difference in how items near to the landmark were encoded, because the biases were specifically in the direction of the landmark displacement, which was entirely unpredictable at the time of encoding. Equally, the biases could not be a consequence of proximity of items in memory to the location of the landmark at the time of retrieval, because this location was also randomized with respect to displacement direction. In contrast, biases in the direction of displacement are fully compatible with an account in which observers remember the relative deviation of items from the landmark, and a model in which this allocentric memory provides an additional, independent source of information for item localization provided an excellent quantitative account of both the biases and the enhancements in precision associated with proximity to the landmark (Fig 4B&4C).

Other than the systematic localization shift in the conflict condition (LM-SHIFT)—which was well characterized by our optimal integration model—we observed no consistent biases in localization due to the presence of the landmark in any of our tasks. However, several previous papers have reported biases, both attractive and repulsive, linked to visual landmarks, as well as fixation and attended, non-fixated locations [32–41]. For example, in a task in which participants were required to make a pointing movement to the location of a single flashed target in the presence of a continuous visual landmark, Diedrichsen and colleagues [32] found that movement endpoints were both repulsed from the location of the landmark and less variable in its vicinity. However, in a similar condition to our LM-ENCODE, in which the landmark was only present during the encoding stage, they observed the presence of the same systematic biases without the improvement in precision. This suggests that the systematic biases they observed are independent of the spatially-specific improvements in precision that occur for items near a landmark. The absence of consistent landmark-related biases in the present experiments may be a consequence of preventing eye movements, ensuring both landmarks and stimuli were equally eccentric, and confining responses to the stimulus circle, all of which would tend to minimize the impact of attentional spatial distortions. Some dynamical models of working memory predict attraction or repulsion between items in memory depending on their separation [42–44], but we would not expect the same principles to apply to the landmark, which as discussed above does not appear to itself be stored in memory.

Our experimental manipulations compared recall in the presence and absence of a landmark object. This allowed us to quantify the performance changes resulting from adding a new source of allocentric information to the scene, irrespective of whether allocentric information was also encoded in the LM-ABSENT condition. One possibility is that participants encoded item locations relative to other elements that remained visible throughout the trial, i.e. the screen edges or the fixation spot. Although we elected not to obscure these elements (removing the fixation spot would have made it impractical to control eye movements), we think a contribution of this relative information to our egocentric estimate is unlikely. In our task, the reliability of allocentric information diminished rapidly with distance from the landmark: indeed, the localization of memoranda more than 4.7° of visual angle (46° on the circle) from the landmark received negligible benefit from allocentric information (< 5% change in precision from no-landmark performance). This renders the distance from the memoranda to the screen edges (min 6.5°) or the fixation dot (6°) too far to exert any meaningful influence on localization. Previous studies have attempted to estimate a distance threshold beyond which allocentric information no longer has a significant influence, based on qualitative comparisons of conditions with different spatial separations [30,32,45]. Our approach enabled us to identify and quantify a continuous change in the reliability of relative cues that occurs as the distance from the landmark increases.

The format in which allocentric information is extracted from the array and stored in memory cannot be unambiguously determined from our experiments. The simplest account of our results would posit an internal representation of the vector connecting each memory item with the landmark. However, it is possible that other static elements in the participant’s surroundings, or overarching geometric principles such as the fact all stimuli were displayed in the vertical plane of the monitor (defining an observer-independent coordinate frame), influence the representation format also. These issues have been explored primarily in the context of navigation and large-scale spatial cognition [46–49]. The present design could in future be extended to examine corresponding principles in VSWM, for example by presenting two or more landmarks in a single memory array.

Competition in encoding information within a feature dimension has been linked to the normalization of neural population activity [50], and this model has been successful in accounting for set size effects [51]. While this neural account of resource limitations has been extended to incorporate multiple feature dimensions, including spatial location [52], no attempt has been made to distinguish between different spatial reference frames. This work has, however, both confirmed and provided new evidence for a privileged role of spatial information in binding object features [53,54]. Evidence for a specific contribution of allocentric information to object binding has been revealed in change detection tasks in which individual item locations (egocentric) or global spatial layout (allocentric) are separately manipulated. Here, even when explicitly informed that location information was irrelevant, performance was compromised by individual changes in spatial position unless allocentric information remained veridical [1,55–58].

While our observation of set size effects on the precision of allocentric information suggests a commonality in neural representation with other feature dimensions, relative location information may be unique in that it spans objects rather than being associated with a single object. For this reason it is unclear how object file [53] or slot-based models of VSWM [2,9] would be able to incorporate such spatial information. Our model does not attempt to capture transformations between egocentric and allocentric reference frames (e.g. [59]) and this will be an important direction for future investigation, particularly with respect to the effects of self-motion.

Classically, the division between egocentric and allocentric information has been associated with the neuropsychological distinction between the dorsal and ventral visual processing streams [60,61]. While spatial information is encoded in egocentric coordinates throughout the dorsal pathway, the ventral projections into the inferior temporal cortex represent progressively more complex information about object properties, encoded by neurons with decreasing sensitivity to spatial location [61–64] and little retinotopic organization [13,14]. Contemporary research suggests that, rather than being lost, spatial information along the ventral path is instead increasingly represented in terms of the relations within and between objects in the environment [65–67]. Indeed, neuroimaging studies looking for correlates of allocentric coding have frequently identified higher areas in the ventral stream [68–73] as components of a broader distributed network contributing to allocentric representation [65,71]. Hippocampal structures are also implicated in relative spatial encoding, most clearly in relation to navigation, but with growing evidence for a role in coding visual space [59,71,74–78].

Despite these recent findings, the neural coding of allocentric space remains far more poorly understood than egocentric space. We believe the present work provides a computational and experimental framework within which future studies can explore the neural bases of these spatial memory mechanisms.

## Methods

### Participants

39 participants took part in the study in total. All participants gave informed consent, in accordance with the Declaration of Helsinki. The study was approved by the Cambridge Psychology Research Ethics Committee. All participants had normal or corrected-to-normal color vision. Each experiment recruited new participants, ensuring all were naïve to the aims of the experiment. Three subjects failed to understand the task and were excluded from analysis (one in Exp 2; two in Exp 3). This left 12 participants in Experiment 1 (age range: 18–28; mean: 24±3; 4 male, 8 female), 12 in Experiment 2 (age range: 20–34; mean: 26±4; 5 male, 7 female), and 12 in Experiment 3 (age range: 19–30; mean: 25±4; 1 male, 11 female). Sample sizes were preselected based on pilot experiments and reports of previous studies examining spatial recall [6,52,79]. Recruiting new participants for each experiment had the advantage of providing multiple internal replications of our key results.

### Experimental design

#### Experiment 1

In the first experiment we examined whether the absence (LM-ABSENT) or presence (LM-PRESENT) of a visual landmark influenced spatial working memory performance. Participants began each trial by fixating a central dot for 500 ms. A sample array of 1, 2 or 4 colored disks (diameter 1° of visual angle, on an invisible circle with a radius of 6°) was then presented for 1000 ms (Fig 1). The colors of the memory items were chosen from a set of four (color [L, a, b]; yellow [50, 20, 80], pink [50, 80, 20], purple [50, 20, –40], green [50, –40, 20]), selected to have maximally distinctive hues in CIELAB colorspace. The sample array was followed by a 1000 ms blank delay period, after which the fixation dot changed to the color of one of the items in the sample array (the *target*). Participants used a computer mouse to indicate the remembered location of the target. First, a mouse cursor appeared at the location of the fixation dot. Once participants moved the mouse cursor >2° from fixation, a response disk (white) appeared on the invisible circle and participants used the mouse to move this disk to the target location. They made their response by clicking on the disk.

LM-ABSENT and LM-PRESENT conditions were identical, apart from a dark gray ([10, 0, 0]) landmark disk (1.5° diameter) that appeared on the invisible circle 500 ms before the sample array in the LM-PRESENT condition and persisted until after participants made their response. Participants were informed that on some trials the gray disk would be present, but that they would only be asked to recall the location of the colored disks and they could think of the gray disk as a background object. The spatial location of the landmark was selected randomly on the circle, while the location of the target stimulus on each trial was randomly assigned such that across each experimental block an equal number of targets occurred at 12 angular bins around the circle relative to the landmark. This was to ensure that all distances from the landmark were approximately equally sampled within each block. Locations of the remaining memory items were randomly assigned, with the constraint that items were separated by at least 15° on the circle (to prevent overlap). There was no such constraint on the landmark, enabling memory items to occasionally overlap it. However, as the landmark was both larger and situated “behind” the memory items, in these cases both landmark and memory items remained visible.

Participants were instructed to maintain fixation at the center of the screen and gaze position was monitored online at 1000 Hz using an infrared eye tracker (Eyelink 1000, SR Research). Trials with eye-movements prior to the response cue were aborted, and a new trial initiated. Both conditions, and all three set sizes, were interleaved. To facilitate later analysis there were six times as many trials in LM-PRESENT (216 per set size) as LM-ABSENT (36 per set size). Participants completed 6 blocks of 126 trials, for a total of 756 trials, taking approximately 1.5 hours.

#### Experiment 2

The second experiment proceeded identically to Experiment 1, with a few notable exceptions. Only the largest set size was tested (4 items) and, in addition to LM-ABSENT and LM-PRESENT, two new conditions were included. The LM-GAP condition was identical to LM-PRESENT except that the landmark was removed at the start of the memory delay, returning at the same time as the response cue. In the LM-ENCODE condition the landmark was removed at the start of the memory delay and did not reappear. Because all four conditions were interleaved, participants did not know during the presentation of the sample array whether the landmark would be present during the delay or response. Participants completed a total of 216 trials in each of the LM-PRESENT, LM-GAP, and LM-ENCODE conditions, and 72 trials in LM-ABSENT. Trials were divided into 6 blocks of 120 trials, for a total of 720 trials, taking approximately 1.5 hours.

#### Experiment 3

The third experiment was identical to Experiment 2 with a few exceptions. The LM-ENCODE condition was removed and replaced with a new condition, LM-SHIFT, which was identical to LM-GAP except that the landmark reappeared in a new position, displaced 6° on the circle randomly clockwise or counter-clockwise from its original location. There were a total of 216 trials in the LM-PRESENT, LM-GAP and LM-SHIFT conditions and 36 trials in LM-ABSENT. Participants completed 6 blocks of 114 trials, for a total of 684 trials, taking approximately 1.5 hours.

### Analysis

We calculated the median angular deviation (a measure of response bias) and the median absolute angular deviation (a measure of response variability) between the response and the target for each condition and, in conditions with a landmark, for different landmark-target distances. For display purposes, we summarized data into 24 partially overlapping bins, separated by 15° and encompassing data from ±15°.

#### Ideal observer model

We modeled localization responses as arising from an optimal integration of two independent estimates of target location: an *egocentric* estimate ${\hat{x}}_{ego}$ that is normally distributed with mean *μ*_*ego*_ and precision (inverse variance) *P*_*ego*_, and an *allocentric* estimate ${\hat{x}}_{allo}$ that is normally distributed with mean *μ*_*allo*_ and precision *P*_*allo*_.

The precision of the egocentric estimate, *P*_*ego*_, does not depend on the landmark location and is a free parameter of the model. The precision of the allocentric estimate declines exponentially with distance *d* between the landmark and the stimulus,

$$P_{allo}=A_{max}\;\exp\left(-A_{scale}\cdot d\right)$$

where *A*_*max*_ and *A*_*scale*_ are free parameters capturing the peak precision and the rate at which precision declines with distance, respectively. For landmark-absent conditions we set *P*_*allo*_ to zero.

The mean of the egocentric estimate of location, *μ*_*ego*_, is in all cases equal to the true location of the stimulus, *x*. This is also true for the mean of the allocentric estimate, except in Exp 3 where the landmark is shifted through a displacement *s* during the delay; in this case the allocentric estimate follows the shifted landmark, i.e. *μ*_*allo*_ = *x* + *s*.

A maximum likelihood estimate (${\hat{x}}_{MLE}$) is obtained by weighting each of the individual estimates by their precision [28,80]. As a result, ${\hat{x}}_{MLE}$ is normally distributed with mean,

$$\mu_{MLE}=\frac{P_{ego}\cdot \mu_{ego}+P_{allo}\cdot \mu_{allo}}{P_{ego}+P_{allo}},$$

and precision,

$$P_{MLE}=P_{ego}+P_{allo}.$$

In generating a response, we allow for a certain proportion of *lapse* trials in which the response is randomly (uniformly) distributed relative to the target location. So, the response distribution is given by,

$$p({\hat{x}}_{resp})=(1-p(lapse))\phi ({\hat{x}}_{resp};\mu_{MLE},P_{MLE})+p(lapse)\frac{1}{2\pi },$$

where *p*(*lapse*) is a free parameter and *ϕ*(*x*;*μ*,*P*) is the von Mises distribution function evaluated at *x* with mean *μ* and precision *P* (we use a von Mises, or circular normal, function because the response space is circular, to ensure the response distribution integrates to one; however, in practice, values of *P*_*MLE*_ that fit the data were always sufficiently high that the von Mises was indistinguishable from a Gaussian with the same mean and precision).

The model was fit to each participant’s data using maximum likelihood obtained by a nonlinear optimization algorithm (*fmincon* in MATLAB). We placed bounds on the free parameters as follows: *P*_*ego*_, [0, ∞]; *A*_*max*_, [0, ∞]; *A*_*scale*_, [0, 500]; *p*(*lapse*), [0, 1]. Tests for effects of experimental condition on model parameters were carried out by comparing models in which the relevant parameter was shared between conditions versus models with independent parameter values for each condition.

Monte-Carlo simulation was used to generate predictions of median absolute angular deviation and median angular deviation to facilitate comparison with behavioral data.

#### Model with cost

To ascertain whether encoding allocentric information decreased precision of egocentric memory, we also examined an extended model with an additional cost parameter (*C*). Here we replaced *P*_*ego*_ with two parameters corresponding to egocentric precision in the presence ($P_{ego}^{LM}$) or absence ($P_{ego}^{NO-LM}$) of a landmark, related by

$$P_{ego}^{LM}=P_{ego}^{NO-LM}(1-C),$$

where *C* is bounded in the range [0, 1]. The inclusion of this cost parameter enabled egocentric precision to be reduced when the landmark was present. This model had five free parameters: $P_{ego}^{NO-LM}$, *A*_*max*_, *A*_*scale*_, *p*(*lapse*), *C*. To test for a cost of landmark encoding we compared this model to the unextended model described above. We used the Akaike Information Criterion with correction for finite sample sizes (AICc) for model comparison. This criterion typically incorporates a smaller penalty for additional parameters than the Bayesian Information Criterion (BIC). Using AICc meant that the addition of a cost parameter was relatively unlikely to be rejected, making it a conservative test of the hypothesis that the landmark conferred no cost.

## Supporting information

S1 Text Testing for an effect of landmark presence at retrieval. Experiment 2 revealed that the presence of a landmark during encoding alone (LM-ENCODE) was not sufficient to induce a landmark effect. This indicates that the benefit of the landmark is not due to items near the landmark capturing attention and being preferentially encoded (leading to greater precision). We are grateful to an anonymous reviewer for suggesting an additional control experiment, in which the landmark is present only at retrieval (LM-RETRIEVE). This experiment tested whether the presence of the landmark at the time of recall conveys a benefit to items whose locations in memory are in its vicinity, for example by directing internal attention to the location in memory corresponding to the landmark.
Twelve new participants (age range: 20–30; mean: 24±4; 3 male, 9 female) took part in Experiment 2B (with 2 additional subjects excluded for not understanding the task), which comprised three interleaved conditions: LM-ABSENT and LM-PRESENT, which were identical to the corresponding conditions in Exp 2, and the new LM-RETRIEVE condition (S1A Fig). In this condition the landmark appeared at a random position on the circle at the same time as the probe color (matching the timing of the landmark’s re-appearance in the LM-GAP condition of Exp 2). Participants completed a total of 210 trials in each of the LM-PRESENT and LM-RETRIEVE conditions, and 105 trials in LM-ABSENT. Trials were divided into 5 blocks of 105 trials, for a total of 525 trials, taking approximately 1 hour.
In the LM-RETRIEVE condition we found no evidence for a spatially-specific improvement in the precision of recall for items in the vicinity of the landmark (S1B Fig). A reduced model in which there was no allocentric signal in this condition provided a better fit to data than the full optimal integration model (ΔAICc = 39.86). This contrasted with the LM-PRESENT condition where, as for the other experiments in this study, excluding the allocentric component made the model worse (ΔAICc = 68.74). These results demonstrate that the presence of a landmark solely during retrieval was insufficient to generate the improvements observed in LM-PRESENT (or LM-GAP, Exp 2) conditions.
As in the other experiments in this study we found no evidence for a cost to precision of egocentric encoding due to the presence of the landmark (model with cost on LM-PRESENT performed worse, ΔAICc = 24.1). We additionally tested whether the appearance of the landmark in the LM-RETRIEVE condition reduced egocentric precision, perhaps by acting as an attentional distractor. We found weak evidence against this (model with cost on LM-RETRIEVE performed worse, ΔAICc = 2.65).
(DOCX)

S2 Text Investigating the cost parameter across experiments. For each of the three experiments reported in the paper, and the supplementary experiment 2B, model comparison supported models in which the precision of egocentric information was unaffected by the presence or absence of a landmark in preference to models in which the presence of the landmark conveyed a cost to egocentric precision. To further validate this result and assess how any potential cost varied between participants, we pooled the cost parameters obtained from models incorporating a cost of landmark presence fit to data from Experiments 2, 2B and 3. Importantly, in the main analysis the cost parameter was constrained to always be positive, in order that the model comparison provided a one-tailed test at the group level of the hypothesis that the presence of the landmark made egocentric precision worse. Here we allowed cost to take on negative values also, so as to fairly assess how the estimates varied between participants. The resulting distribution of cost parameter values is shown in S2A Fig. We found that when cost was incorporated as a free parameter in the model, it took up a relatively broad range of values, both positive and negative, across participants, with an average that was negative but close to zero (mean ± SEM: –0.070 ± 0.030; median: –0.063%). This is consistent with the findings of formal model comparison which indicated no evidence at the group level for a (positive) cost of landmark presence, implying that allocentric estimates of location do not compete with egocentric estimates for representation in memory. To further evaluate these data, we considered what cost we would expect if the converse were true, i.e. were egocentric and allocentric representations to compete for the same working memory resources. We reasoned that in this case the addition of a landmark would double the number of competing location representations, because the egocentric representation of each memory stimulus would be supplemented by an allocentric representation encoding its location relative to the landmark. The proportionate decrease in egocentric precision (the cost) should therefore be comparable to that observed when the number of memory items is doubled, which would also be expected to double the number of representations in memory. An estimate of this effect is available from Exp 1, calculated as the ratio of *P*_*ego*_ estimates obtained at set size 2 and set size 1, or alternatively at set size 4 and set size 2. This method produced predicted costs of 0.24 ± 0.04 and 0.20 ± 0.04, respectively (S2B Fig). The large majority of participants (97% versus 2:1 estimate; 92% versus 4:2 estimate) had estimated cost parameters below these predictions, further strengthening the evidence for independence of allocentric and egocentric working memory stores. (Note that data from Exp 1 was excluded from the pooling of cost estimates specifically to avoid circularity in this comparison).
(DOCX)

S1 Fig Experiment 2B. **(A)** The paradigm for experiment 2B, including the new LM-RETRIEVE condition to investigate whether landmarks present only during response convey any benefit to recall **(B-C)** Mean variability in memory recall across participants for LM-RETRIEVE (B) and LM-PRESENT (C) conditions (with LM-ABSENT shown on the right in blue). There was no apparent influence of the visual landmark when it was only visible during response (LM-RETRIEVE). Predictions of the best-fitting model are overlaid.
(TIF)

S2 Fig Meta-analysis of cost parameter estimates. (A) Histogram of estimates of cost (proportionate decrease in *P*_*ego*_) due to presence of the landmark in Experiments 2, 2B and 3 (based on LM-PRESENT and LM-ABSENT conditions; all set size 4). **(B)** The mean cost (red) across participants is compared to the proportionate change in *P*_*ego*_ associated with doubling the number of memory items (Exp 1). Under the hypothesis of shared resources, these estimates should be equal. Instead, the mean cost of adding a landmark is small in magnitude compared to increasing set size, and in the opposite direction (i.e. a minor benefit of the landmark).
(TIF)

S3 Fig Experiment one. **(A-C)** Average bias (+ve, CW) in location recall for set sizes 1, 2 and 4 respectively, with the best fitting model overlaid. There were no consistent biases related to distance from the landmark.
(TIF)

S4 Fig Experiment two. Parameter estimates for the best-fitting model. Parameters for egocentric precision (A) and lapse rate (D) were common to all three conditions, while the best fitting model for LM-Encode had no allocentric components. While there was no difference in the maximum allocentric precision between LM-GAP and LM-PRESENT (B), there was a small difference in the allocentric scale (C).
(TIF)

S5 Fig Experiment three. Parameter estimates for the best-fitting model. Parameters for egocentric precision (A) and lapse rate (D) were common to all three conditions. While parameters for maximum allocentric precision were shared between LM-GAP and LM-PRESENT, the maximum allocentric precision in the LM-SHIFT condition was decreased (B). The three conditions were best fit with differing allocentric scale parameters (C).
(TIF)
