# Supplementary material for: Independent working memory resources for egocentric and allocentric spatial information
Source: PLoS Comput Biol. 2019 Feb 21;15(2):e1006563. doi: 10.1371/journal.pcbi.1006563 (PMC6400418; doi:10.1371/journal.pcbi.1006563)
Supplement: S1 Text — Experiment 2 revealed that the presence of a landmark during encoding alone (LM-ENCODE) was not sufficient to induce a landmark effect. This indicates that the benefit of the landmark is not due to items near the landmark capturing attention and being preferentially encoded (leading to greater precision). We are grateful to an anonymous reviewer for suggesting an additional control experiment, in which the landmark is present only at retrieval (LM-RETRIEVE). This experiment tested whether the presence of the landmark at the time of recall conveys a benefit to items whose locations in memory are in its vicinity, for example by directing internal attention to the location in memory corresponding to the landmark. Twelve new participants (age range: 20–30; mean: 24±4; 3 male, 9 female) took part in Experiment 2B (with 2 additional subjects excluded for not understanding the task), which comprised three interleaved conditions: LM-ABSENT and LM-PRESENT, which were identical to the corresponding conditions in Exp 2, and the new LM-RETRIEVE condition (S1A Fig). In this condition the landmark appeared at a random position on the circle at the same time as the probe color (matching the timing of the landmark’s re-appearance in the LM-GAP condition of Exp 2). Participants completed a total of 210 trials in each of the LM-PRESENT and LM-RETRIEVE conditions, and 105 trials in LM-ABSENT. Trials were divided into 5 blocks of 105 trials, for a total of 525 trials, taking approximately 1 hour. In the LM-RETRIEVE condition we found no evidence for a spatially-specific improvement in the precision of recall for items in the vicinity of the landmark (S1B Fig). A reduced model in which there was no allocentric signal in this condition provided a better fit to data than the full optimal integration model (ΔAICc = 39.86). This contrasted with the LM-PRESENT condition where, as for the other experiments in this study, excluding the allocentric component made the model worse (ΔAICc = 68.74) [file pcbi.1006563.s001.docx]

**Supporting Text S1. Testing for an effect of landmark presence at retrieval.**

Experiment 2 revealed that the presence of a landmark during encoding alone (LM-ENCODE) was not sufficient to induce a landmark effect. This indicates that the benefit of the landmark is not due to items near the landmark capturing attention and being preferentially encoded (leading to greater precision). We are grateful to an anonymous reviewer for suggesting an additional control experiment, in which the landmark is present only at retrieval (LM-RETRIEVE). This experiment tested whether the presence of the landmark at the time of recall conveys a benefit to items whose locations in memory are in its vicinity, for example by directing internal attention to the location in memory corresponding to the landmark.

[Figure S1 HERE]

**Figure S1. Experiment 2B. (A)** The paradigm for experiment 2B, including the new LM-RETRIEVE condition to investigate whether landmarks present only during response convey any benefit to recall **(B-C)** Mean variability in memory recall across participants for LM-RETRIEVE (B) and LM-PRESENT (C) conditions (with LM-ABSENT shown on the right in blue). There was no apparent influence of the visual landmark when it was only visible during response (LM-RETRIEVE). Predictions of the best-fitting model are overlaid.

Twelve new participants (age range: 20–30; mean: 24±4; 3 male, 9 female) took part in Experiment 2B (with 2 additional subjects excluded for not understanding the task), which comprised three interleaved conditions: LM-ABSENT and LM-PRESENT, which were identical to the corresponding conditions in Exp 2, and the new LM-RETRIEVE condition (S1A Fig). In this condition the landmark appeared at a random position on the circle at the same time as the probe color (matching the timing of the landmark’s re-appearance in the LM-GAP condition of Exp 2). Participants completed a total of 210 trials in each of the LM-PRESENT and LM-RETRIEVE conditions, and 105 trials in LM-ABSENT. Trials were divided into 5 blocks of 105 trials, for a total of 525 trials, taking approximately 1 hour.

In the LM-RETRIEVE condition we found no evidence for a spatially-specific improvement in the precision of recall for items in the vicinity of the landmark (S1B Fig). A reduced model in which there was no allocentric signal in this condition provided a better fit to data than the full optimal integration model (ΔAICc = 39.86). In contrast, a model with no allocentric signal for the LM-PRESENT condition performed worse (ΔAICc = 68.74). These results demonstrate that the presence of a landmark solely during retrieval was insufficient to generate the improvements observed in LM-PRESENT (or LM-GAP, Exp 2) conditions.

As in the other experiments in this study we found no evidence for a cost to precision of egocentric encoding due to the presence of the landmark (model with cost on LM-PRESENT performed worse, ΔAICc = 24.1). We additionally tested whether the appearance of the landmark in the LM-RETRIEVE condition reduced egocentric precision, perhaps by acting as an attentional distractor. We found weak evidence against this (model with cost on LM-RETRIEVE performed worse, ΔAICc = 2.65).
