# Supplementary material for: Independent working memory resources for egocentric and allocentric spatial information
Source: PLoS Comput Biol. 2019 Feb 21;15(2):e1006563. doi: 10.1371/journal.pcbi.1006563 (PMC6400418; doi:10.1371/journal.pcbi.1006563)
Supplement: S2 Text — For each of the three experiments reported in the paper, and the supplementary experiment 2B, model comparison supported models in which the precision of egocentric information was unaffected by the presence or absence of a landmark in preference to models in which the presence of the landmark conveyed a cost to egocentric precision. To further validate this result and assess how any potential cost varied between participants, we pooled the cost parameters obtained from models incorporating a cost of landmark presence fit to data from Experiments 2, 2B and 3. Importantly, in the main analysis the cost parameter was constrained to always be positive, in order that the model comparison provided a one-tailed test at the group level of the hypothesis that the presence of the landmark made egocentric precision worse. Here we allowed cost to take on negative values also, so as to fairly assess how the estimates varied between participants. The resulting distribution of cost parameter values is shown in S2A Fig. We found that when cost was incorporated as a free parameter in the model, it took up a relatively broad range of values, both positive and negative, across participants, with an average that was negative but close to zero (mean ± SEM: –0.070 ± 0.030; median: –0.063%). This is consistent with the findings of formal model comparison which indicated no evidence at the group level for a (positive) cost of landmark presence, implying that allocentric estimates of location do not compete with egocentric estimates for representation in memory. To further evaluate these data, we considered what cost we would expect if the converse were true, i.e. were egocentric and allocentric representations to compete for the same working memory resources. We reasoned that in this case the addition of a landmark would double the number of competing location representations, because the egocentric representation of each memory stimulus would be supplemented by an allocentric representat [file pcbi.1006563.s002.docx]

**Supporting Text S2. Investigating the cost parameter across experiments**

For each of the three experiments reported in the paper, and the supplementary experiment 2B, model comparison supported models in which the precision of egocentric information was unaffected by the presence or absence of a landmark in preference to models in which the presence of the landmark conveyed a cost to egocentric precision. To further validate this result and assess how any potential cost varied between participants, we pooled the cost parameters obtained from models incorporating a cost of landmark presence fit to data from Experiments 2, 2B and 3. Importantly, in the main analysis the cost parameter was constrained to always be positive, in order that the model comparison provided a one-tailed test at the group level of the hypothesis that the presence of the landmark made egocentric precision worse. Here we allowed cost to take on negative values also, so as to fairly assess how the estimates varied between participants. The resulting distribution of cost parameter values is shown in Figure S2A.

We found that when cost was incorporated as a free parameter in the model, it took up a relatively broad range of values, both positive and negative, across participants, with an average that was negative but close to zero (mean ± SEM: –0.070 ± 0.030; median: –0.063%). This is consistent with the findings of formal model comparison which indicated no evidence at the group level for a (positive) cost of landmark presence, implying that allocentric estimates of location do not compete with egocentric estimates for representation in memory. To further evaluate these data, we considered what cost we would expect if the converse were true, i.e. were egocentric and allocentric representations to compete for the same working memory resources. We reasoned that in this case the addition of a landmark would double the number of competing location representations, because the egocentric representation of each memory stimulus would be supplemented by an allocentric representation encoding its location relative to the landmark. The proportionate decrease in egocentric precision (the cost) should therefore be comparable to that observed when the number of memory items is doubled, which would also be expected to double the number of representations in memory. An estimate of this effect is available from Exp 1, calculated as the ratio of $P_{ego}$ estimates obtained at set size 2 and set size 1, or alternatively at set size 4 and set size 2. This method produced predicted costs of 0.24 ± 0.04 and 0.20 ± 0.04, respectively (S2B Fig). The large majority of participants (97% versus 2:1 estimate; 92% versus 4:2 estimate) had estimated cost parameters below these predictions, further strengthening the evidence for independence of allocentric and egocentric working memory stores. (Note that data from Exp 1 was excluded from the pooling of cost estimates specifically to avoid circularity in this comparison).

[Figure S2 Here]

**Figure S2. Meta-analysis of cost parameter estimates**. (A) Histogram of estimates of cost (proportionate decrease in $P_{ego}$) due to presence of the landmark in Experiments 2, 2B and 3 (based on LM-PRESENT and LM-ABSENT conditions; all set size 4). **(B)** The mean cost (red) across participants is compared to the proportionate change in $P_{ego}$ associated with doubling the number of memory items (Exp 1). Under the hypothesis of shared resources, these estimates should be equal. Instead, the mean cost of adding a landmark is small in magnitude compared to increasing set size, and in the opposite direction (i.e. a minor benefit of the landmark).
