# Supplementary figures and images for: Independent working memory resources for egocentric and allocentric spatial information
Source: PLoS Comput Biol. 2019 Feb 21;15(2):e1006563. doi: 10.1371/journal.pcbi.1006563 (PMC6400418; doi:10.1371/journal.pcbi.1006563)

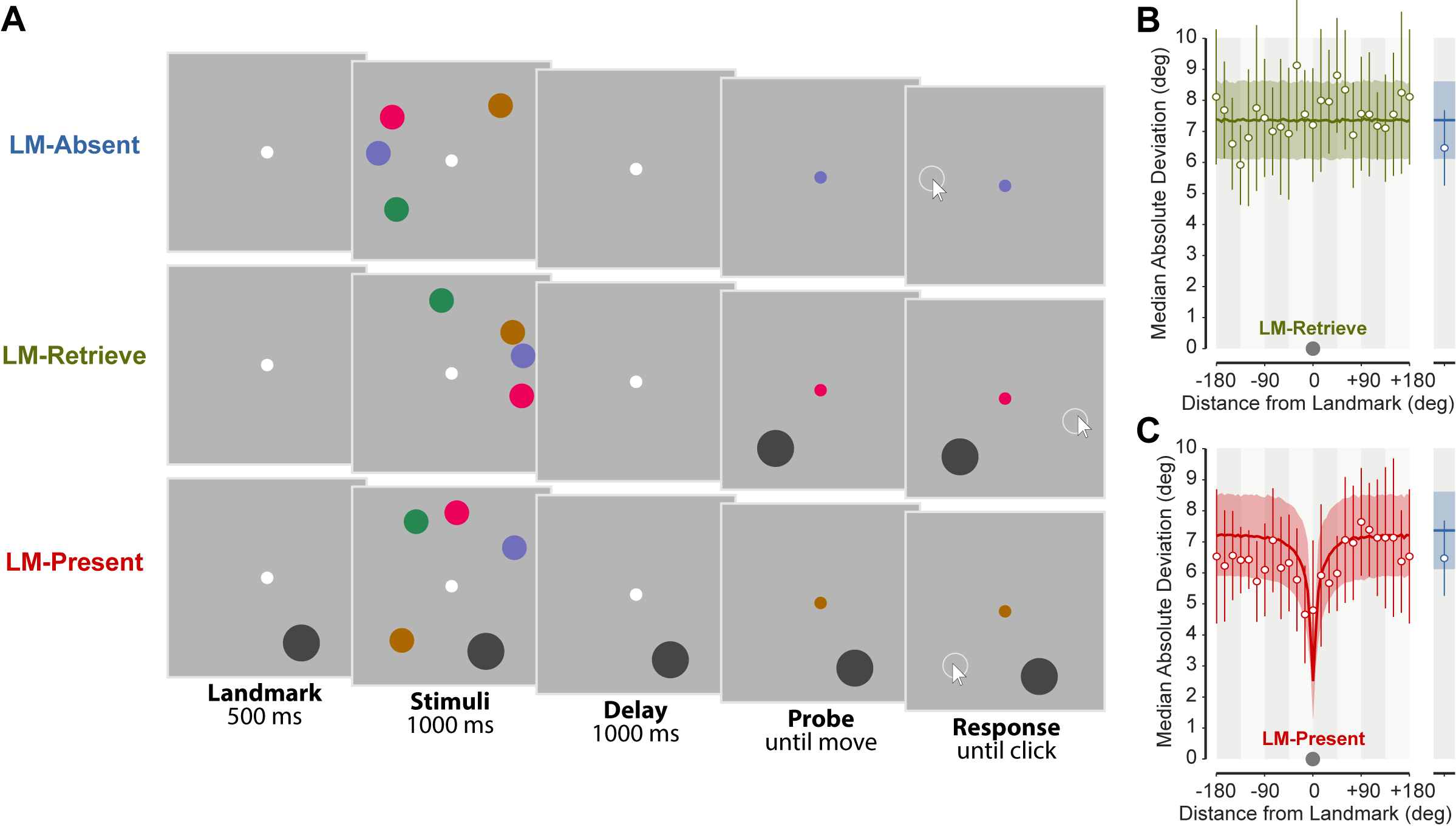

Supplement: S1 Fig — (A) The paradigm for experiment 2B, including the new LM-RETRIEVE condition to investigate whether landmarks present only during response convey any benefit to recall (B-C) Mean variability in memory recall across participants for LM-RETRIEVE (B) and LM-PRESENT (C) conditions (with LM-ABSENT shown on the right in blue). There was no apparent influence of the visual landmark when it was only visible during response (LM-RETRIEVE). Predictions of the best-fitting model are overlaid. (TIF) [file pcbi.1006563.s003.tif]

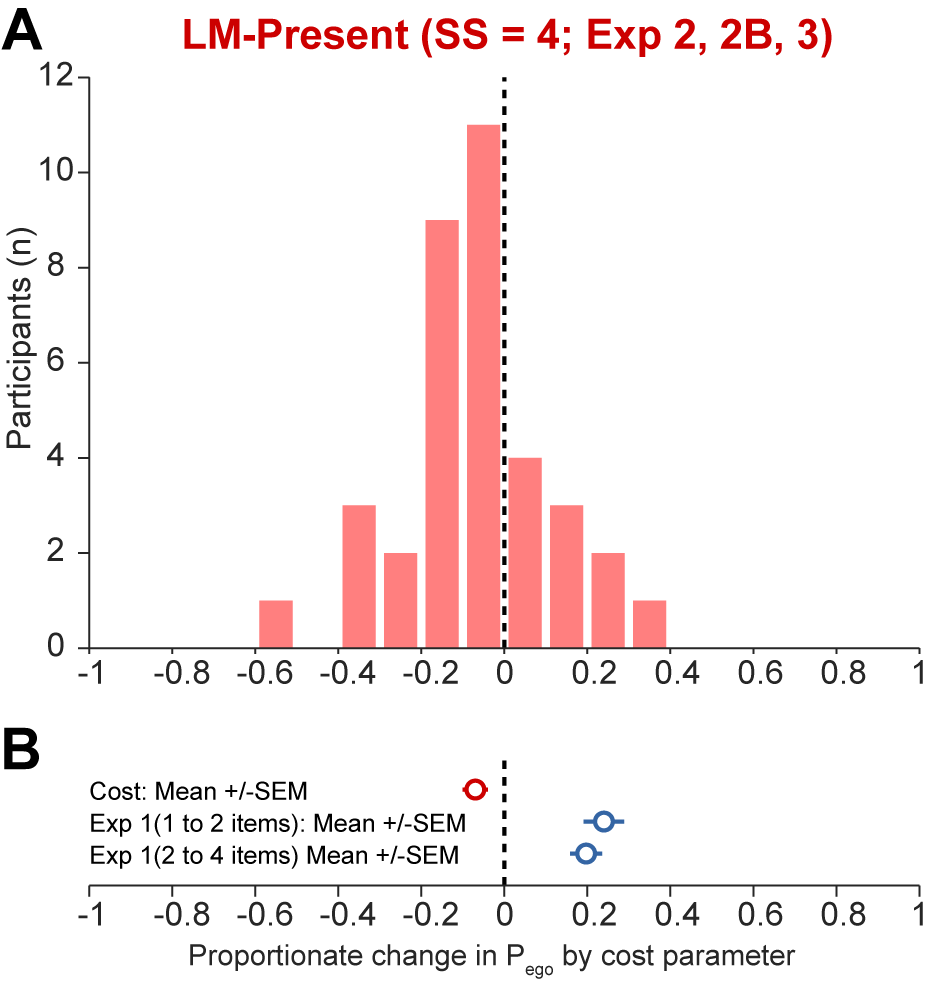

Supplement: S2 Fig — (A) Histogram of estimates of cost (proportionate decrease in Pego) due to presence of the landmark in Experiments 2, 2B and 3 (based on LM-PRESENT and LM-ABSENT conditions; all set size 4). (B) The mean cost (red) across participants is compared to the proportionate change in Pego associated with doubling the number of memory items (Exp 1). Under the hypothesis of shared resources, these estimates should be equal. Instead, the mean cost of adding a landmark is small in magnitude compared to increasing set size, and in the opposite direction (i.e. a minor benefit of the landmark). (TIF) [file pcbi.1006563.s004.tif]

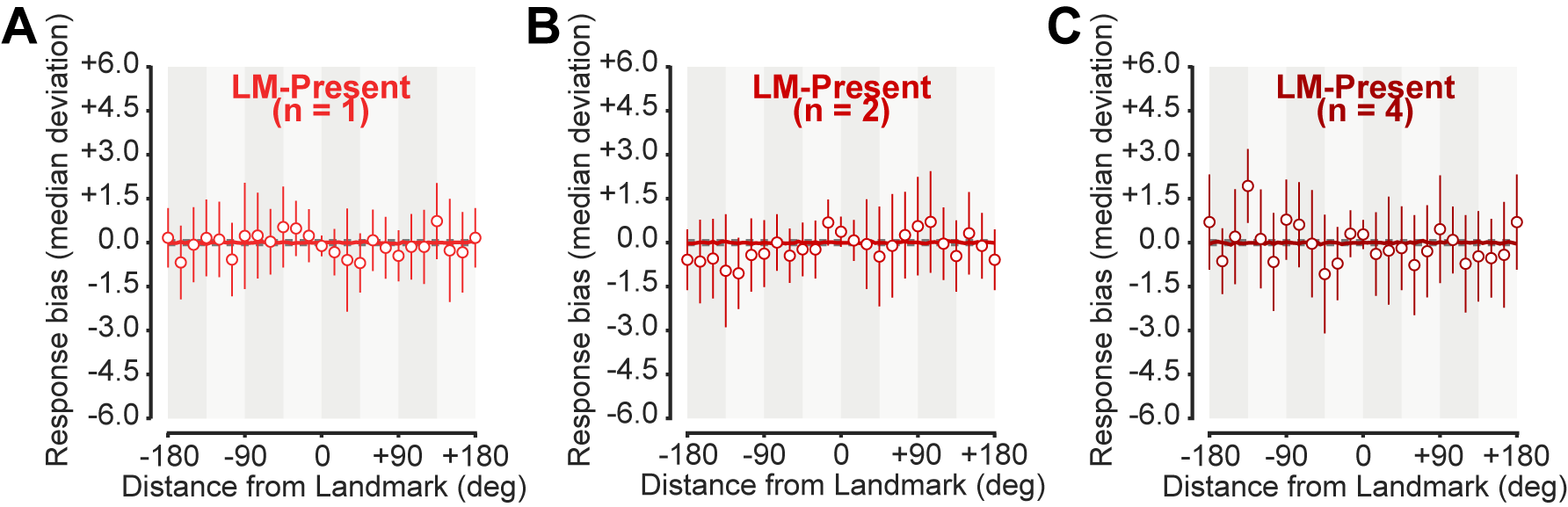

Supplement: S3 Fig — (A-C) Average bias (+ve, CW) in location recall for set sizes 1, 2 and 4 respectively, with the best fitting model overlaid. There were no consistent biases related to distance from the landmark. (TIF) [file pcbi.1006563.s005.tif]

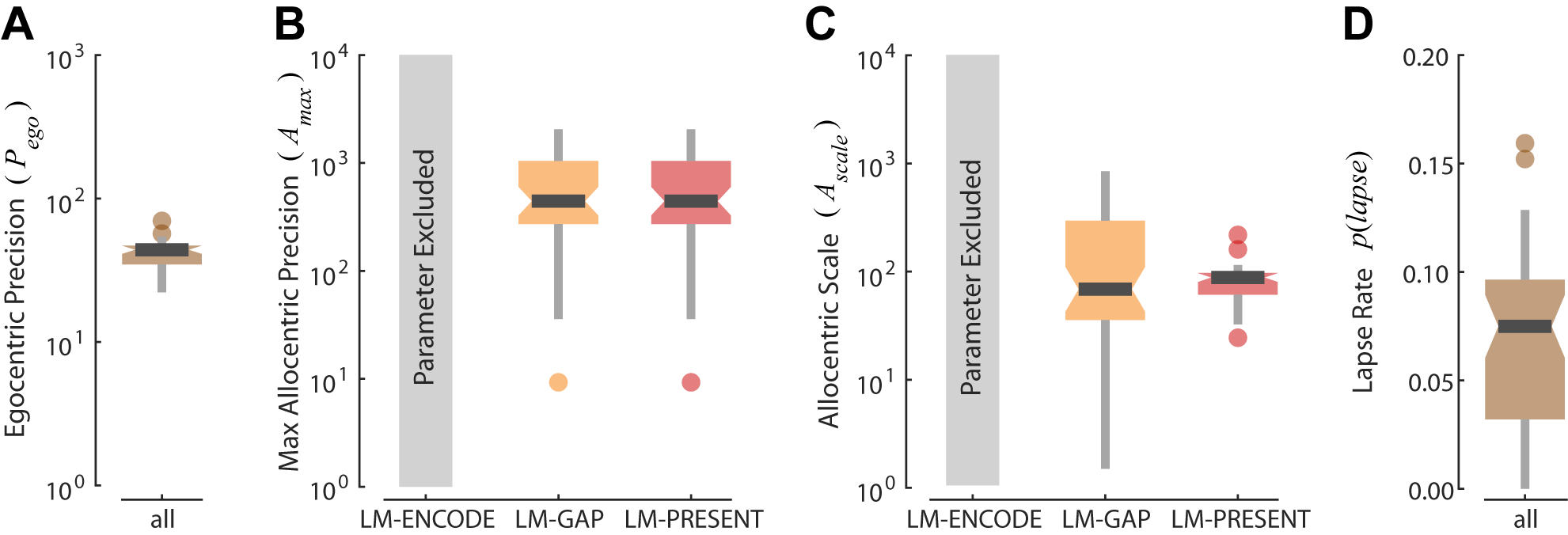

Supplement: S4 Fig — Parameter estimates for the best-fitting model. Parameters for egocentric precision (A) and lapse rate (D) were common to all three conditions, while the best fitting model for LM-Encode had no allocentric components. While there was no difference in the maximum allocentric precision between LM-GAP and LM-PRESENT (B), there was a small difference in the allocentric scale (C). (TIF) [file pcbi.1006563.s006.tif]

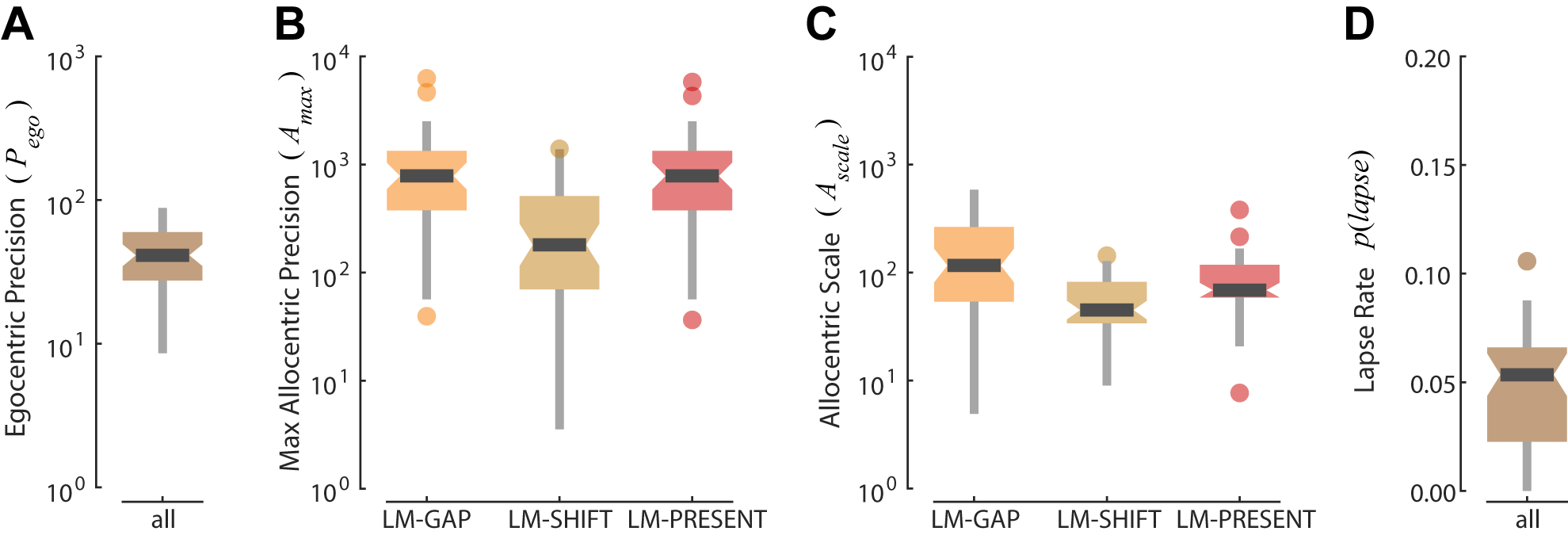

Supplement: S5 Fig — Parameter estimates for the best-fitting model. Parameters for egocentric precision (A) and lapse rate (D) were common to all three conditions. While parameters for maximum allocentric precision were shared between LM-GAP and LM-PRESENT, the maximum allocentric precision in the LM-SHIFT condition was decreased (B). The three conditions were best fit with differing allocentric scale parameters (C). (TIF) [file pcbi.1006563.s007.tif]
